# What Role Does Trabecular Bone Score Play in Chronic Inflammatory Rheumatic Diseases?

**DOI:** 10.3389/fmed.2020.600697

**Published:** 2020-11-30

**Authors:** Barbara Ruaro, Andrea Casabella, Luigi Molfetta, Francesco Salton, Paola Confalonieri, Marco Confalonieri, Elisa Baratella, Antonio De Tanti, Cosimo Bruni

**Affiliations:** ^1^SC Pneumologia, ASUGI, Trieste, Italy; ^2^Research Laboratory and Academic Division of Clinical Rheumatology, Department of Internal Medicine, San Martino Polyclinic Hospital, University of Genoa, Genoa, Italy; ^3^Department of Internal Medicine Di.M.I, Osteoporosis, Bone and Joint Disease Research Center, CROPO, University of Genoa, Genoa, Italy; ^4^Department of Integrated Surgical and Diagnostic Sciences, School of Medical and Pharmaceutical Sciences, University of Genoa, Genoa, Italy; ^5^Department of Radiology, Department of Medicine, Surgery and Health Science, University of Trieste, Trieste, Italy; ^6^Cardinal Ferrari Centre, S. Stefano Rheabilitation, Fontanellato, Italy; ^7^Division of Rheumatology, Department of Experimental and Clinical Medicine, University of Firenze, Florence, Italy

**Keywords:** osteoporosis, rheumatic diseases, Trabecular Bone Score (TBS), Bone Mineral Density (BMD), osteopaenia

## Abstract

Patients suffering from rheumatic inflammatory diseases, e.g., systemic sclerosis, rheumatoid arthritis, and ankylosing spondylitis, are at risk of low bone mass. Dual-energy X-ray Absorptiometry (DXA) is the traditional radiological measurement technique for bone mineral density (BMD). The recently developed trabecular bone score (TBS) enhances the skeletal information provided by standard BMD. It re-analyzes the spatial dynamics of pixel intensity changes in lumbar spine DXA images, defining a quantitative index, characterizing trabecular bone microarchitecture. It has been demonstrated that low TBS values are associated with an increased incidence of fractures in patients with rheumatic diseases. These methods used together for bone damage evaluation can be of value to identify individuals who will potentially fracture. The main scientific literature on the clinical aspects of osteoporosis, including the use of TBS in evaluating this pathology, are herein reported aimed at shedding light on the role trabecular bone score plays in chronic inflammatory rheumatic diseases.

## Highlights

- Patients affected by rheumatic diseases are prone to an increased risk of low bone mass.
- The trabecular bone score provides information on the bone microstructure of patients with rheumatic diseases.
- Patients with rheumatic diseases have lower TBS values than healthy subjects.

## Introduction

The trabecular bone score (TBS) a grayscale measurement of texture which is derived from the evaluation of the experimental variogram obtained from the Dual-energy X-ray Absorptiometry (DXA) images, is a relatively new tool to evaluate bone microarchitecture (1). TBS is an indirect measurement of bone axial microarchitecture, providing information on bone quality, e.g., trabecular quantity, trabecular separation, connectivity density, and Parfitt parameters (1–3). Previous reports proposed a normal TBS value for post-menopausal women of 1.350 or more; conversely, scores between 1.200 and 1.350 were attributed to a partially degraded microarchitecture and a TBS below 1.200,a degraded microarchitecture (3–5). These cut-off points were established by analogy with the three bone mineral density (BMD) categories, i.e., normal bone mass, osteopenia and osteoporosis (OP) (3–5). More recently, Anderson et al. developed reference ranges for TBS suitable for use in a clinical setting in an Australian male cohort: scores equal to, or lower than, 1.003 were considered for the determination of degraded microarchitecture (6). The authors also demonstrated a linear life-time decrease in TBS amongst the males, (whereas the decrease in women is better modeled with a cubic function) (6). The strength of TBS lies in its ability to provide more information on the potential risk of vertebral fractures than the DXA and BMD. Indeed, its use as an adjuvant to the standard DXA exam was approved by both the Food and Drug Administration (FDA) and the European Medicines Agency (EMA) (4, 7, 8). Despite this, to date, there are no guidelines on how to apply it to standard practice, even if the World Health Organization (WHO) established operational definitions of osteoporosis (OP) and osteopenia in postmenopausal Caucasian women (4, 5, 7, 8). These were based on BMD values and aimed at guiding researchers and clinicians in the classification of degrees of bone loss, as early as 1994. Several studies reported that patients with chronic rheumatic inflammatory diseases have a higher OP and osteopenia risk, based on BMD (5, 7, 8). However, several limitations in BMD sensitivity have been reported, such as its low power in determining bone quality (5, 7, 8), an important factor when assessing bone fragility. Indeed, it has recently been demonstrated that bone microarchitecture plays a pivotal role in bone strength. Evidence to date indicates that the TBS bone texture index is able to add further skeletal information to that obtained by the standard BMD (6, 9–11). Several studies confirmed that TBS can discriminate patients with altered bone microstructure and have proposed its use as a clinical-radiological tool in OP diagnosis in patients with chronic inflammatory rheumatic diseases, such as systemic sclerosis (SSc), systemic lupus erythematosus (SLE), rheumatoid arthritis (RA), and ankylosing spondylitis (AS) (12–18).

## TBS and Systemic Sclerosis

SSc is a connective tissue disease characterized by early microvascular impairment, skin, and internal organ fibrosis (19–26). Several studies have also recently demonstrated an increased risk of OP in SSc patients, correlated with multiple factors, i.e., low vitamin D levels (1, 5, 13, 15, 27). Other studies have reported lower BMD and TBS values in SSc patients compared to healthy matched controls (1, 2, 5, 13). Ruaro et al. reported that SSc patients with a “Late” nailfold capillaroscopy pattern had lower TBS values than patients with an “Active” or “Early” pattern (“Late” vs. “Active” and “Early” pattern, *p* < 0.001) (5, 13); whilst no statistically significant difference in BMD values was observed when comparing the three different capillaroscopy patterns (5, 13). The negative correlation between the reduced bone microarchitecture, evaluated by TBS, and the progression of microvascular damage studied by nailfold videocapillaroscopy (NVC), suggested that the microvascular damage in SSc patients is also correlated to bone impairment and other systemic complications (5, 13, 22, 26). Furthermore, these studies confirmed that SSc patients have a higher OP and osteopenia risk associated with the BMD obtained by DXA (1, 2, 5, 13, 15). Various articles report that TBS is an index of bone texture which is able to enhance the skeletal information obtained by the standard BMD, also in SSc patients (2, 5, 13). Indeed, several reports demonstrated that bone microarchitecture plays a pivotal role in bone strength and that TBS can be a clinical tool for OP diagnosis in scleroderma patients (1, 2, 5, 13).

Bone damage may have multi-factorial underlying causes: disability, age, longstanding diseases, low Body Mass Index (BMI), chronic systemic inflammation, low vitamin D levels, and some treatment regimes (1, 2, 5, 13, 27). Numerous authors demonstrated the presence of lower serum 25-hydroxy-vitamin D (25(OH)D) levels in SSc patients than in healthy subjects (HS) (5, 13, 27). Two recent studies have reported that the 25(OH)D level is significantly lower in the “Late” than in the “Active” or “Early” capillaroscopy pattern patients (*p* = 0.002), probably attributable to a reduced vitamin D intestinal absorption (5, 13, 27). The same studies demonstrated that a positive 25(OH)D value correlated with TBS but not with BMD, as also observed by Koumakis et al. (2). Ruaro et al. reported that there were statistically significant lower bone alkaline phosphatase (bone ALP) levels in SSc patients than in HS and that the “Late” pattern patients had lower ALP levels than those with an “Active” or “Early” one, probably due to a reduced turnover and neo-bone formation (5, 13, 27). Furthermore, there was a positive correlation between the TBS and the bone ALP values (*p* < 0.0001) and a negative correlation with the onset of Raynaud's phenomenon in the SSc patients (*p* < 0.01). Conversely, there was no statistically significant correlation between the TBS values and calcium or phosphorus blood levels (5, 13).

In conclusion, all these studies demonstrated that SSc patients run a high risk of having low bone mass and support the importance of evaluating the different aspects of bone architecture with DXA, TBS, and bone parameters, such as vitamin D circulating levels, as part of the periodical clinical assessment (1, 2, 5, 13, 27).

## TBS and Systemic Lupus Erythematosus

Systemic lupus erythematosus (SLE) is a complex systemic autoimmune disease, characterized by a wide spectrum of clinical and serological manifestations (14, 28–30). Recent studies have demonstrated a higher incidence of OP and bone fractures in SLE patients compared to HS (14, 30–36). The bone loss observed in SLE has a multi-factorial etiology, including: systemic inflammation, kidney impairment, nutritional disorders, serological, metabolic, and hormonal factors and maybe also genetic factors and drugs, such as glucocorticoids (GC) (14, 30–40). Moreover, a high prevalence of morphometric vertebral fractures has been observed in SLE patients, despite the fact that 1/3 of them had normal bone density, in line with the hypothesized multi-factorial etiology of fractures in SLE (14, 30–40). Indeed, long-term use of corticosteroids may induce OP in SLE patients by influencing bone turnover (increasing bone resorption and decreasing bone formation), preventing the formation of collagen and osteocalcin, as well as reducing the bone matrix mineralization (14, 30–40). Moreover, numerous cytokines are involved in OP pathogenesis, due to their influence on osteoblast and osteoclast function, i.e., IL-33 (35, 37–39). Vitamin D deficiency may also be a predisposing factor for bone loss in SLE (14, 35, 37–39). Several studies have demonstrated that SLE patients have an increased risk of low bone mass, assessed by DXA and TBS (14, 30–40).

Lai et al. and Ruaro et al. emphasized the important role TBS plays as an innovative and safe diagnostic tool for the quantification of bone quality in chronic and systemic inflammatory rheumatic diseases, such as those observed in SLE. In fact, both studies confirmed that SLE patients have a higher risk of bone loss (osteopenia and OP) and fractures than do HS (34, 40).

Lai et al. analyzed 147 SLE patients and observed that TBS had a higher diagnostic accuracy for vertebral fractures than densitometric measurements (area in the ROC curve for TBS, L spine and left femur BMD: 0.811 vs. 0.737 and 0.605, respectively), supporting the assessment of bone microarchitecture by TBS as an enhancer of the information provided by BMD and showing that TBS identified degraded microarchitecture mainly associated with vertebral fractures in SLE patients (34).

Ruaro et al. were the first to evaluate bone involvement in SLE and compare the results with matched HS, using TBS and DXA (40). They observed that the lumbar spine TBS score was significantly lower in SLE patients than in HS (*p* < 0.001) and that BMD was significantly lower in all areas (the lumbar spine, femoral neck, Ward's triangle, trochanter, and hip) than in HS (*p* < 0.001, for all areas) (40). Furthermore, this study showed that SLE patients had an increased prevalence of 25(OH) vitamin D insufficiency (*p* < 0.001) than HS, as frequently reported in rheumatic diseases (36, 40). Interestingly, SLE patients with previous fractures had statistically significantly lower vitamin D values than those without (*p* < 0.0001) (36, 40).

Another recent study has indicated that supplementation with high vitamin D doses (1.400 IU cholecalciferol per day) and calcium carbonate (1.250 mg per day) for 6 months improved bone mineral density and decreased the osteopenia and OP rates in corticosteroid-treated patients. Most likely, vitamin D can activate osteoblast and bone formation, as well as decrease bone resorption, through the inactivation of osteoclasts (37–39).

Therefore, we consider the early identification of OP and osteopenia is a must in a SLE subjects with “fragile bones,” as is the set-up of specific diagnostic-therapeutical strategies. Enhanced knowledge of bone pathophysiology, coupled to progress in pharmaceutical development, has provided the opportunity to make early identification of subjects at high risk of fragility fractures and to start preventive therapy for fractures.

## TBS and Rheumatoid Arthritis

Rheumatoid arthritis (RA) is a chronic inflammatory disease which affects the joints, with progressive and destructive consequences and may also have extra-articular manifestations (41, 42).

These manifestations could be related to the localization of the rheumatoid process in other tissues, i.e., serosa (pericardium, pleura), skin (rheumatoid nodules) or medical therapy complications, such as OP (41, 42).

Some studies reported that the frequency of OP in women with RA ranged from 30 to 50%, depending on the areas assessed by the DXA, this datum was also confirmed in males (43); osteopenia had a prevalence of about 80% (43). Several factors may lead to adverse effects on bone mass and to an increased risk of fracture in RA patients, i.e., reduced sun exposure leading to low serum 25(OH) vitamin D levels, reduced physical activity, proximal muscle atrophy due to a sedentary lifestyle, prolonged use of GC and disease-induced bone mass reduction. Indeed, RA patients tend to develop early OP and are prone to fragility fractures (2, 9, 12, 43–45).

Several studies have demonstrated decreased BMD values using DXA but only a few studies have made use of TBS in RA patients. Breban et al. proposed evaluating the diagnostic TBS as a complement to the BMD on DXA or as an independent risk factor for vertebral fractures (VF) in populations on GC or anti-TNF therapy (43). TBS values were lower in patients on GC compared to those who were not (*p* = 0.0001). Furthermore, TBS was significantly lower in VF patients compared to those without fractures (*p* = 0.0001) and it had a better discrimination value to predict VF in RA patients than the lumbar spine BMD alone (43, 45).

TBS was assessed in patients with RA and AS and compared with healthy subjects (HS) in a case-control study by Toussirot et al. (44). Moreover, they made a prospective examination of the changes in the BMD values of lumbar spine and hip, along with the TBS values whilst on anti-TNF drugs. The study enrolled 30 RA and 30 AS patients not on GC and a comparative HS group of 50 subjects. TBS values from L2 to L4 were measured and the BMD and T-Score were evaluated at the hip in RA patients. They observed that both values were significantly lower in the RA subjects than in the HS (*p* = 0.005). Interestingly, the subgroup of 20 patients on anti-TNF (8 RA and 12 AS patients) monitored for 2 years during the perspective phase of the study, showed a significant increase in the lumbar spine BMD values (+6.3 and + 2.4%, respectively, for RA and AS). However, TBS significantly decreased in RA patients, whilst it remained stable in AS patients, which may be explained by the different influences these drugs have on the bone. The final analysis of the study showed that TBS in RA patients on anti-TNF allows for a greater discrimination of the population at lumbar spine fracture risk, increasing the percentage of the population to be treated with anti-osteoporotic therapies compared to the data provided by DXA alone (44).

Koumakis et al. and Ruaro et al. compared RA, SSc, and HS using TBS and DXA in conjunction (2, 5). Koumakis et al. observed no significant difference in the average lumbar spine TBS values between RA and SSc patients; similarly, BMD at the lumbar spine, femoral neck, and total hip did not differ among the three groups. The fracture prevalence was similar in the RA and SSc groups (29.2 vs. 33.3%, respectively, *p* = 0.682). The TBS values did not differ between RA and HS (*p* = 0.128), despite lower cumulative and daily GC dose (*p* < 0.0001). Furthermore, no association between GC and TBS was observed in the RA group (2). Ruaro et al. selected 98 RA patients on a daily GC dose of <5 mg/day and 60 HS. They observed that 78/98 of the RA group (80%) had bone loss at DXA and BMD. BMD was significantly lower in RA patients than in the control group (*p* < 0.001) (5). Similarly, lumbar spine TBS was significantly lower in RA patients than in HS (*p* < 0.001). Furthermore, their study confirmed that 25(OH)-D serum levels were statistically significantly lower in RA patients than in HS (*p* < 0.001) (5).

Similar results were confirmed by Casabella et al., who evaluated 108 females affected by RA and 60 HS. They performed DXA and TBS at the level of the lumbar spine (L1-L4) and evaluated the serum 25(OH) vitamin D concentrations, for all patients. The lumbar spine TBS score was significantly lower in RA sufferers compared to HS (*p* < 0.001). Moreover, subjects with RA had lower 25(OH) vitamin D concentrations than HS (*p* < 0.04) (45).

## TBS and Ankylosing Spondylitis

AS is a chronic inflammatory form of arthritis involving the axial skeleton (46, 47), affecting the spinal vertebrae and sacroiliac joints, causing debilitating pain and loss of mobility. It has been proposed that the sites of attachment of the ligaments or tendons to the bone, known as entheses, are the major target of the inflammatory, traumatic, and degenerative pathological changes occurring in AS (46–48). Enthesitis is believed to play a primary role in the ligament calcification process, which leads to pain. It can cause reduced flexibility of the spine and eventually complete loss of spinal mobility, destruction as well as ankylosis (fusion) of the spine and sacroiliac joints.

New bone formation, which includes the development of syndesmophytes and ankylosis of the spine, is almost pathognomonic for AS (47–51).

The altered new bone formation in the vertebral cortical area and the impairment of trabecular bone at the level of the vertebral body increase the risk of both OP and VF. Furthermore, these bone alterations and ligament ossification modify the BMD data and falsely increase lumbar spine BMD values (41, 47, 51).

Since AS mainly affects young men and BMD gives falsely increased values, there is often a delay in prevention and/or treatment of OP in this condition. Therefore, appropriate bone assessment to determine bone strength, microstructure and any ossifications is a must to start correct treatment in routine practice (17, 18, 47–51).

Toussirot et al. assessed TBS in 30 patients with RA and AS compared the data to those of 50 HS, also including 20 patients who had been on anti-TNF drugs for 2 years (44). The lumbar spine BMD did not differ between AS and HS patients, whilst there was a decrease in the hip T-score in AS patients (*p* = 0.02) (44). Several studies reported a 25% OP prevalence in AS patients and from 10 to 43% radiographic vertebral fractures (17, 18, 47–51).

Ivanova et al. evaluated the relationship between physical function, disease activity, spine mobility, and bone parameters, TBS and BMD, in AS patients (50). The study concluded that lumbar BMD can be affected by osteoproliferation and that, despite this, AS patients have a lower TBS score than HS. Moreover, more evident alterations are also reported in bone microarchitecture in older patients, without significant differences between genders. There was an inverse correlation between the mobility scores and the three bone parameters (TBS, BMD, and T-score femoral), showing a relationship between the state of skeletal health and vertebral functional deterioration (50).

Recently, Richards et al. reported the first analysis of TBS for fracture prediction as an incident event in AS: TBS was shown to independently predict major osteoporotic and clinical spine fractures in AS, whatever the FRAX score (48). Similar predictive power was confirmed by Nam et al., who studied 215 AS patients (75.8% males) and reported that TBS could predict the risk of major osteoporotic fractures. They also stated that TBS is not influenced by spinal osteo-proliferation in AS patients, even in those with advanced spinal changes (49, 51).

These data could overcome the bias derived from previous reports with falsely elevated DXA values at the level of the lumbar spine in AS (49–51). This finding is most likely due to the fact that DXA measures only the quantity and not the *quality* of the bone, therefore confirming its limitations for fracture prediction in this patient group.

## TBS and Other Rheumatic Diseases

Recent studies reported that TBS could also be useful in other rheumatic diseases, such as osteoarthritis (OA) and polymyalgia rheumatica (PMR) (52–54). Kolta et al. enrolled 1,254 menopausal women and evaluated 727 of them for 6-years. Patients with lumbar OA had a higher BMD than those without lumbar OA at the lumbar spine, but not at the hip. Conversely, spine TBS did not differ between patients with or without lumbar OA (*p* = 0.70). Interestingly, there was a negative correlation between spine TBS and BMD at all sites and age (*p* < 0.0001) (52). In conclusion, numerous studies have shown that whilst BMD values can be overestimated in patients with OA, TBS evaluations do not seem to be affected by OA changes in the lumbar spine. Furthermore, as TBS is mildly impacted by OA, it could be a better predictor of fracture than spine BMD (52, 53).

Several studies reported that TBS could also be a supplementary tool to discriminate osteoporotic fractures in postmenopausal patients with PMR (54). Kim et al. compared BMD, TBS and the frequency of VF in patients with PMR, RA, or HS. The researchers demonstrated that in PMR patients had a significantly higher VF frequency than RA and HS patients (*p* = 0.017). The average TBS in PMR patients was significantly lower than that of RA and HS patients (*p* < 0.001). Their multivariate analysis demonstrated that a lower TBS is associated with VF in PMR patients (*p* = 0.043). In conclusion, TBS is a promising technique, even if further studies should be carried out to clarify the role this technique plays in other specific rheumatic disorders (see Table 1).

**Table 1:** Milestones in the study of bone microarchitecture analyzed by trabecular bone score (TBS) in rheumatic diseases.

**Authors**	**Study**	**Rheumatologic**	**Control**	**Summary of**
	**type**	**population**	**population**	**results**
Kounakis et al. (2)	CS	138 RA, W	227 mHC	TBS did not differ between controls and RA patients, despite lower cumulative, and daily glucocorticoid (GC) dose. No association between GC and TBS was found in RA
Choi et al. (9)	CS	279 RA, pW	NA	The TBS was negatively correlated with the cumulative dose of glucocorticoids (GCs), but not with the disease activity score for 28 joints (DAS28) or erythrocyte sedimentation rate
Casabella et al. (12)	P	55 RA, W	55 mHC	Most of RA patients (80%) had lower BMD than control group Lumbar spine TBS was found significantly lower in RA patients compared with mHC Positive correlation between the TBS and relative skeletal mass index (RSMI) in RA patients
Ruaro et al. (13)	P	60 RA, W	60 mHC	The BMD values and the T-score measured on the vertebral column, the femoral neck, and the whole femur were significantly lower in RA patients than those in the control group Lumbar spine TBS was found significantly lower in RA patients compared with mHC
Kim et al. (16)	P	100 RA, W aged ≥50	NA	Twenty-six patients were revealed to have moderate to severe vertebral fractures There was a modest negative correlation between fracture risk assessment score (FRAX) and TBS. There was no correlation between FRAX and L-spine BMD
Breban et al. (43)	P	185 RA, W	NA	T-scores were significantly lower in patients with VFs than in patients without VFs, the largest difference being observed at femoral neck. TBS was significantly lower in patients with VFs vs. without VFs
Toussirot et al. (44)	CC, P	30 RA, W	50 mHC	RA patients had lower BMD, lower T score, and lower TBS at the hip compared to mHC Under anti-TNFa, in patients with RA, TBS score decreased
Casabella et al. (45)	P	108 RA, W	60 mHC	78 RA patients (80%) presented a bone loss that was significantly lower when compared with mHC. Lumbar spine TBS score was significantly lower in RA patients compared with mHC
Ruaro et al. (5)	P	84 SSc, pW	60 mHC	TBS and BMD were significantly lower in SSc patients than in mHC TBS values were found to be lower in SSc with a “Late” nailfold videocapillaroscopy (NVC) pattern, compared with the “Active” or “Early” patterns
Ruaro et al. (13)	P	60 SSc pW	60 mHC	The SSc patients showed higher Dkk-1 serum levels than mHC SSc patients, showing the “Late” NVC pattern had statistically higher Dkk-1 serum levels than patients with either the “Active” or “Early” patterns Only in the “Late” NVC pattern group of SSc patients was there a significant negative correlation between Dkk-1 and TBS values
Ruaro et al. (40)	P	40 SLE, W	40 mHC	The lumbar spine TBS score was statistically significantly lower in SLE patients than in mHC
Casabella et al. (14)	P	70 SLE, W	65 mHC	Lumbar spine TBS score and BMD value were found significantly lower in SLE patients compared with CNT
Caparbo et al. (18)	P	73 AS, M	52 mHC	No difference was observed in lumbar spine BMD in AS patients and CNT, but total hip BMD and TBS were lower in AS patients
Nam et al. (49)	P	215 AS, 75.8% M	NA	TBS, hip BMD, and L-spine lateral BMD showed comparably high areas under the curve for predicting FRAX-major osteoporotic fractures. TBS negatively correlated with modified Stoke AS Spine Score (mSASSS) in both male and female patients
Toussirot et al. (44)	P	30 AS, 27 M	50 mHC	Hip T score in patients with AS was also decreased Lumbar spine (LS) BMD did not differ between patients and mHC, whileTBS was lower in AS compared to HC LS and hip BMD increased after 24 months under anti-TNFa, with significant changes at the spine in patients with AS, TBS progressively increased
Ivanova et al. (50)	P	50 AS, 27 M, 23 W	NA	Lumbar spine BMD can be erroneously influenced by osteoproliferation, unlike the TBS and TBS T-score. The limitations in spinal mobility predicted abnormal results for these two TBS parameters
Wildberger et al. (51)	P	51 AS, M	NA	axSpA men with and without syndesmophytes have lower results compared to the normal population regarding hip BMD, spine TBS, and spine BMD except for men with syndesmophytes who have a normal BMD spine T-score
Boussonalim et al. (17)	P	95 axSpA	NA	Lumbar BMD was positively correlated with TBS, while disease duration, disease activity score and serum PTH levels were negatively correlated with TBS More than half of the patients with a BMD level above −2.5 T-score had a low TBS value
Kolta et al. (52)	P	1,254 patients (including patients with OA), pW		Patients with lumbar osteoarthritis had an BMD higher than those without lumbar osteoarthritis at the lumbar spine, but not at the hip. In contrast, spine TBS was not different between patients with and without lumbar osteoarthritis
Kim et al. (54)	CS, P	53 PMR pW	106 mHC	The mean TBS of patients with PMR was significantly lower than those in CNT TBS could be a supplementary tool for discriminating osteoporotic fractures in postmenopausal patients with PMR

*AS, ankylosing spondylitis; axSpA, axial spondylarthritis; BMD, bone mass density; CC, case-control study; CS, cross-sectional study; GC, glucocorticoids; LS, lumbar spine; M, male patients; mHC, age- and gender-matched healthy controls; NA, not applicable; OA, osteoarthritis; P, prospective study; pW, postmenopausal women; PMR, polymyalgia rheumatica; RA, rheumatoid arthritis; SLE, systemic lupus erythematosus; SSc, systemic sclerosis; TBS, trabecular bone score; VF, vertebral fractures; W, women*.

## Conclusions

Various imaging techniques able to provide direct information on trabecular bone microarchitecture are currently available, such as magnetic resonance and computed tomography. However, their use in clinical practice is hampered by the fact that they are expensive, not always readily available and can examine only the peripheral bone area (18).

There is increasing evidence that TBS values are associated with the incidence of fractures in rheumatic diseases. TBS provides data on trabecular bone microarchitecture, as it is an index of bone texture and enhances the information obtained by the standard BMD. DXA and TBS, used together for bone damage evaluation, can be of value to identify individuals with potentially increased risk on bone fractures and, therefore, guide treatment decisions, particularly in patients with complicated diseases such as rheumatic inflammatory disorders (1, 5, 13, 50, 54, 55).

In conclusion, current evidence supports the use of an integrated assessment plan with TBS and BMD in conjunction, offering advantages in clinical practice over the use of BMD alone when facing the assessment of bone status, also in rheumatic diseases (1, 5, 50, 54–57). Moreover, future research agenda should aim at further studies investigating into the role of TBS as an outcome measure in the evaluation of anti-osteoporotic treatment efficacy.

## Author Contributions

BR, AC, and CB: made substantial contributions to the conception and design of the work, the acquisition, analysis, and interpretation of data, drafting the work and revising it critically for important intellectual content, and the final approval of the version to be published. LM, FS, PC, MC, and AD: made substantial contributions to the analysis and interpretation of data and final approval of the version to be published. All authors contributed to the article and approved the submitted version.

## Conflict of Interest

The authors declare that the research was conducted in the absence of any commercial or financial relationships that could be construed as a potential conflict of interest.
